# Processing of fMRI-related anxiety and bi-directional information flow between prefrontal cortex and brain stem

**DOI:** 10.1038/s41598-021-01710-8

**Published:** 2021-11-16

**Authors:** Gert Pfurtscheller, Katarzyna J. Blinowska, Maciej Kaminski, Andreas R. Schwerdtfeger, Beate Rassler, Gerhard Schwarz, Wolfgang Klimesch

**Affiliations:** 1 Institute of Neural Engineering, Graz University of Technology, Graz, Austria; 2 Nalecz Institute of Biocybernetics and Biomedical Engineering, Polish Academy of Sciences, Ks. Trojdena 4, 02-109 Warsaw, Poland; 3 Faculty of Physics, University of Warsaw, Pasteura 5, 02-093 Warsaw, Poland; 4 Institute of Psychology, University of Graz, Graz, Austria; 5 Carl-Ludwig-Institute of Physiology, University of Leipzig, Leipzig, Germany; 6 Department of Anaesthesiology and Intensive Care Medicine, Medical University of Graz, Graz, Austria; 7 Centre of Cognitive Neuroscience, University of Salzburg, Salzburg, Austria

**Keywords:** Anxiety, Anxiety, Computational neuroscience, Neuro-vascular interactions

## Abstract

Brain–heart synchronization is fundamental for emotional-well-being and brain–heart desynchronization is characteristic for anxiety disorders including specific phobias. Recording BOLD signals with functional magnetic resonance imaging (fMRI) is an important noninvasive diagnostic tool; however, 1–2% of fMRI examinations have to be aborted due to claustrophobia. In the present study, we investigated the information flow between regions of interest (ROI’s) in the cortex and brain stem by using a frequency band close to 0.1 Hz. Causal coupling between signals important in brain–heart interaction (cardiac intervals, respiration, and BOLD signals) was studied by means of Directed Transfer Function based on the Granger causality principle. Compared were initial resting states with elevated anxiety and final resting states with low or no anxiety in a group of fMRI-naïve young subjects. During initial high anxiety the results showed an increased information flow from the middle frontal gyrus (MFG) to the pre-central gyrus (PCG) and to the brainstem. There also was an increased flow from the brainstem to the PCG. While the top-down flow during increased anxiety was predominant, the weaker ascending flow from brainstem structures may characterize a rhythmic pacemaker-like activity that (at least in part) drives respiration. We assume that these changes in information flow reflect successful anxiety processing.

## Introduction

Brain–heart synchronization represents a coherent or synchronous behavior of cardiovascular, respiratory, and neural systems in a frequency range around ~ 0.1 Hz, supporting the close mutual interaction between brain and heart introduced by Claude Bernard about 150 years ago (details see the review)^1^. In contrast, brain–heart desynchronization characterizes anxiety disorders (AD), the most common mental health problem^2^. About 15% of patients seem to report severe claustrophobic reactions during fMRI scanning^3^, and 1–2% of fMRI examinations have to be aborted due to claustrophobia^4^. In a recently published review article about brain–heart desynchronization in AD, Tumati et al.^5^ observed neuro-cardiac desynchronization that was accompanied by a decrease in serotonergic and noradrenergic activity with abnormal connectivity of different neural networks as well as vascular and neural BOLD components at ~ 0.1 Hz. Although it is quite evident that being placed in an MRI machine induces unwellnes, fear or anxiety and in some cases even claustrophobic reactions, little is known how different levels of state anxiety affect BOLD signals, it is reasonable to assume that healthy young MRI participants, without any former scanner experience, felt increased anxiety at least in initial sessions. Such an increase in anxiety with a subsequent decline was reported by Chapman et al.^6^. Of note, these authors also mentioned an anxiety increase towards the end of the session. In accordance with these findings, in a related study, the majority of healthy MRI-naïve participants who showed declining anxiety levels had elevated anxiety in the first resting state. A few individuals displayed no decline or even increased anxiety in the final resting state^7^.

The decline of anxiety in healthy MRI-naïve participants can be seen as a model for successful anxiety processing. In the present study, we used a within-person anxiety classification with elevated anxiety in the first resting states and low or no anxiety in the last resting states. Related studies on elevated fMRI-induced anxiety, which focused on phase coupling between beat-to-beat interval (RRI) and BOLD oscillations, reported slow neural and vascular BOLD components; note, the former lagging the latter, and most interestingly provided first evidence for the existence of a pacemaker-like oscillatory source in the brainstem^8,9^. So far, an open question refers to the direction of the information flow between the cortex and the brainstem and whether a possible rhythmic source in the brainstem contributes to the decline in anxiety. To investigate this question, the calculation of the Directed Transfer Function (DTF) based on the Granger causality principle^10^ was overtaken. DTF is robust to noise and common feeding effect and has been extensively applied to estimate EEG, ECoG, and local field potential (LFP) connectivity patterns^11–13^.

An important structure for brain–heart interaction is the brainstem incorporating the cardiovascular and respiratory centers, the ascending reticular activating system (ARAS^14^), and specific nuclei responsible for secretion of serotonin and noradrenaline^5^. Unfortunately, neural activity in the brainstem cannot be studied directly but only indirectly by recording BOLD signals. BOLD oscillations are complex signals comprising different components^15,16^ that often are associated with oscillatory neural activity^17,18^ but also with movements.

The goal of this communication is first to analyze the bi-directional information flow between the prefrontal cortex (PFC) and the human brainstem, which is associated with a decline of anxiety (i.e., successful anxiety processing) in healthy MRI participants by calculating the DTF^11^. Second, we aim to show that anxiety is associated with an increase in top-down control, which is reflected by a directed increase in causal coupling between the PFC and the brainstem. Third, using ffDTF we sought further evidence for a pacemaker-like activity of an autonomous oscillator^19^ originating in the brainstem.

For such an approach, RRI and respiratory signals, in addition to BOLD recordings from PFC and brain stem, are helpful. Ongoing slow fluctuations of cardiac intervals and neural activity contain important information about brain–heart interaction. For this interaction, the brainstem and the PFC play a crucial role. We selected the left middle frontal gyrus (MFG/ROI 7), a large area which—among many other functions—regulates amygdala activity in AD^20^ and the left pre-central gyrus (PCG/ROI 1) as part of the somatomotor network affected in AD^5^. In the brainstem, two structures in the pons were selected, a more rostral part (ROI 93) and a more caudal part (ROI 103). These two ROIs showed relatively stable results on cardiac-BOLD coupling^9^. Note that the locus coeruleus (LC) in the pons is involved in physiological responses to stress, anxiety, and depression, similar to the raphe nuclei in the brainstem. Both nuclei, which are associated with the control of noradrenergic and serotonergic activity, are involved in the processing of anxiety^5^.

## Methods

### Study approval

All participants gave informed written consent to the protocol of the study, which was approved by the local Ethics Committee at the University of Graz (number: GZ. 39/75/63 ex 2013/14). Our research was performed in accordance with the ethical standards laid down in the 1964 Declaration of Helsinki.

### Study design

A prerequisite to study successful anxiety processing is to have a sample of healthy subjects each with few resting states and clearly declining anxiety state. From this sample two groups were distinguished, one with elevated anxiety (HA samples) at the beginning of the experiment and another with no or low anxiety (LA samples) at the end. The base for this was an fMRI study with four resting states including ECG recording during scanning and within-scanner questionnaire.

### Experimental paradigm

The experimental design consisted of two sessions (each lasting about 45 min) separated by a break of about 50 min and four resting states each lasting about 5 min, two (R1, R2) in the first and two in the second session (R3, R4). Within-scanner questionnaires were carried out just before the resting states R1 and R3 and just after the resting states R2 and R4. Filling out each questionnaire took approximately 5 min. The time span between the first R1 and last R4 resting state was about 2 h.

State anxiety (AS) was assessed with the state-trait anxiety and depression inventory (STADI). The STADI^21^ is an instrument constructed to assess both state and trait aspects of anxiety and depression^22^. The items were presented on a screen within the scanner and were answered via a trackball.

### Participants

A total of N = 23 participants (12 female, 22 right-handed) between 19 and 34 years (M = 24, SD = 3.2 years) took part in the fMRI study. Participants were naïve to the purpose of the study, had no former MRI experience, and were without any record of neurological or psychiatric disorders (assessed by self-report). From the 23 participants only 14 displayed the required AS decline. Four exhibited a medium AS level with increased anxiety to the end (non-successful anxiety processing), and the rest showed e**i**ther strong artifacts in the ECG or only minimal changes in AS (AS change ≤ 2). From the remaining 14 subjects, the HA sample contained subjects with elevated anxiety (AS ≥ 17) and the LA sample (AS ≤ 15) with weak or no anxiety.

### Physiological signal recording and RRI time courses

ECG and respiration were recorded inside the scanner. The sampling rate was 400 Hz. QRS detection and subsequent computation of beat-to-beat interval (RRI) time series was performed using fMRI plug-in for EEGLAB^23^. To further improve RRI signals, the Kubios HRV Premium Package^24^ was used. For further details see^7^.

### Resting state fMRI and ROI selection

Functional images were acquired with a 3 T scanner (Magneton Skyra, Siemens, Erlangen, Germany) using a multiband GE-EPI sequence^25^ with a simultaneous six-band acquisition with TE/TR = 34/871 ms, 52° flip angle, 2 × 2 × 2 mm^3^ voxel size, 66 contiguous axial slices (11 × 6), acquisition matrix of 90 × 104 and a FOV of 180 × 208 mm^2^. For further details see. Finally, the AAL^26^ atlas was used to extract time courses for specified ROI in the left pre-central gyrus (PCG, ROI 1, 3526 voxel), left middle frontal gyrus (MFG, ROI 7, 4860 voxel), and left cerebellum/ brain stem (ROI 93, 1894 voxel, ROI103, 1887 voxel).

### Computing of causal coupling and statistic

The causal coupling between signals was studied by means of Directed Transfer Function (DTF), which is an extension of the Granger causality principle to the multivariate case^13^. DTF is robust to noise, the common feeding effect, and above all, it allows to identify reciprocal connections, which is not possible in the case of bivariate connectivity measures such as, e.g., coherence or phase-locking index^12^.

DTF was calculated in the multivariate autoregressive (MVAR) model framework, which was fitted simultaneously to all considered six signals^11,27^. Here we applied the ffDTF, full frequency version of DTF, normalized in such a way that the normalizing factor in the denominator is independent of frequency:

$${\text{ffDTF}}_{ji} (f) = \frac{{\left| {H_{ji} (f)} \right|^{2} }}{{\sum\nolimits_{f} {\sum\limits_{m = 1}^{k} {\left| {H_{im} (f)} \right|^{2} } } }},$$

where *H*_*ji*_(*f*) is a transfer function of the MVAR model, *f* is frequency. The ffDTF describes the causal influence of channel *j* on channel *i* at frequency *f* and takes values in the range [0, 1].

In order to find prevailing trends in couplings between considered channels, we have integrated the ffDTF(*f*)s in bands of interest, and we have averaged the results over epochs. In this way *C*_*ji*_, the coupling strength in the given frequency band, was found for each pair of channels.

The statistical procedure of finding significant differences in average coupling values *C*_*ji*_ for HA and LA in the chosen frequency band was based on the bootstrap approach. The theoretical distribution of *C*_*ji*_ is unknown; therefore, we based statistical inference on the comparisons of its values with the distributions of the corresponding values for surrogate data obtained by means of bootstrap. The following bands were studied: 0.05–0.15 Hz, 0.1–0.2 Hz and 0.2–0.4 Hz. Only results from the 0.05–0.15 Hz band are reported.

The hypotheses were:

- H0: *D*_*ji*_ = 0, no difference *D*_*ji*_ ï exists between HA and LA samples; and alternatively:
- H1: *D*_*ji*_ ≠ 0,

where *D*_*ji*_ = *C*_*ji*_^HA^ − *C*_*ji*_^LA^ and *C*_*ji*_^HA^ is the connectivity estimated for HA sample and *C*_*ji*_^LA^ is the connectivity estimated for the LA sample. The distribution of *D*_*ji*_ corresponding to the null hypothesis was obtained in the following bootstrap procedure:

A common pool of subjects from both sample values was created. From this pool we:

1. Randomly select (with repetitions) a subset of 14 subjects (8 epochs each) to be marked as type1.
2. Randomly select (with repetitions) another subset of 14 subjects (8 epochs each) to be marked as type2.
3. Compute *D*_*ji*_^boot^ = *C*_*ji*_^type1^ − *C*_*ji*_^type2^ for every *i*, *j*.
4. Repeat the steps one to three 10,000 times, save every obtained *D*_*ji*_^boot^ value. After that step we got from the collected values an empirical distribution of *D*_*ji*_.
5. From the distributions obtained in step 4 we find connections for which original values of connectivity difference *D*_*ji*_ lie outside the assumed confidence range (here: 95%). We considered these connections as significant.

The same procedure was applied for testing distinctions between inflows and outflows from/to the given channel.

## Results

### Successful anxiety processing

**Figure 1:**
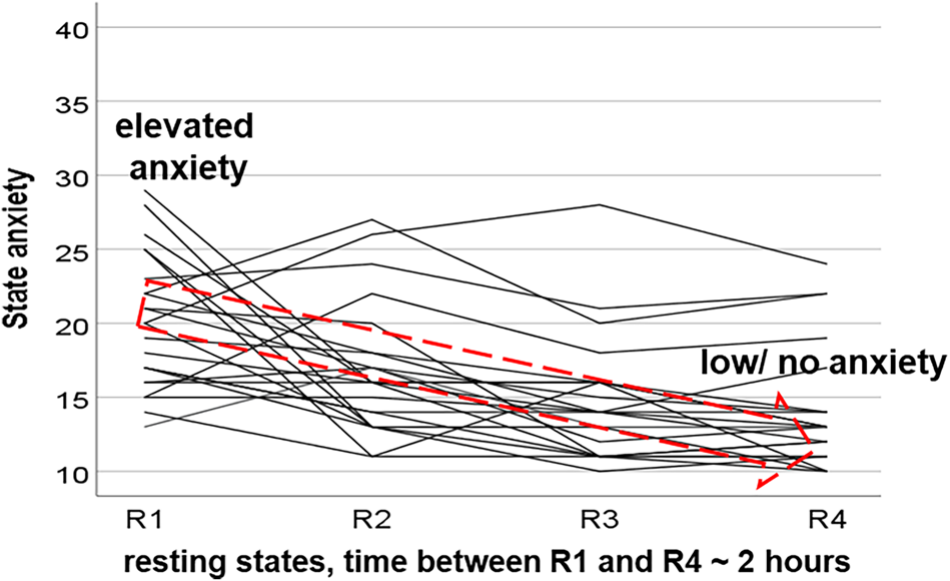
Trajectories of AS in 23 healthy fMRI-naïve young subjects. The red arrow with stippled lines indicates the sample of subjects with significantly decreased anxiety with AS = 21.9 ± 4.1 (mean ± SD) for the HA sample and AS = 12.4 ± 1.5 for the LA sample.

The selected individuals displayed declining anxiety, which may indicate successful anxiety processing (Fig. 1). Noteworthy, although the majority of subjects showed an apparent anxiety decline, for some subjects, we observed increased anxiety across resting states.

### Causal coupling

**Figure 2:**
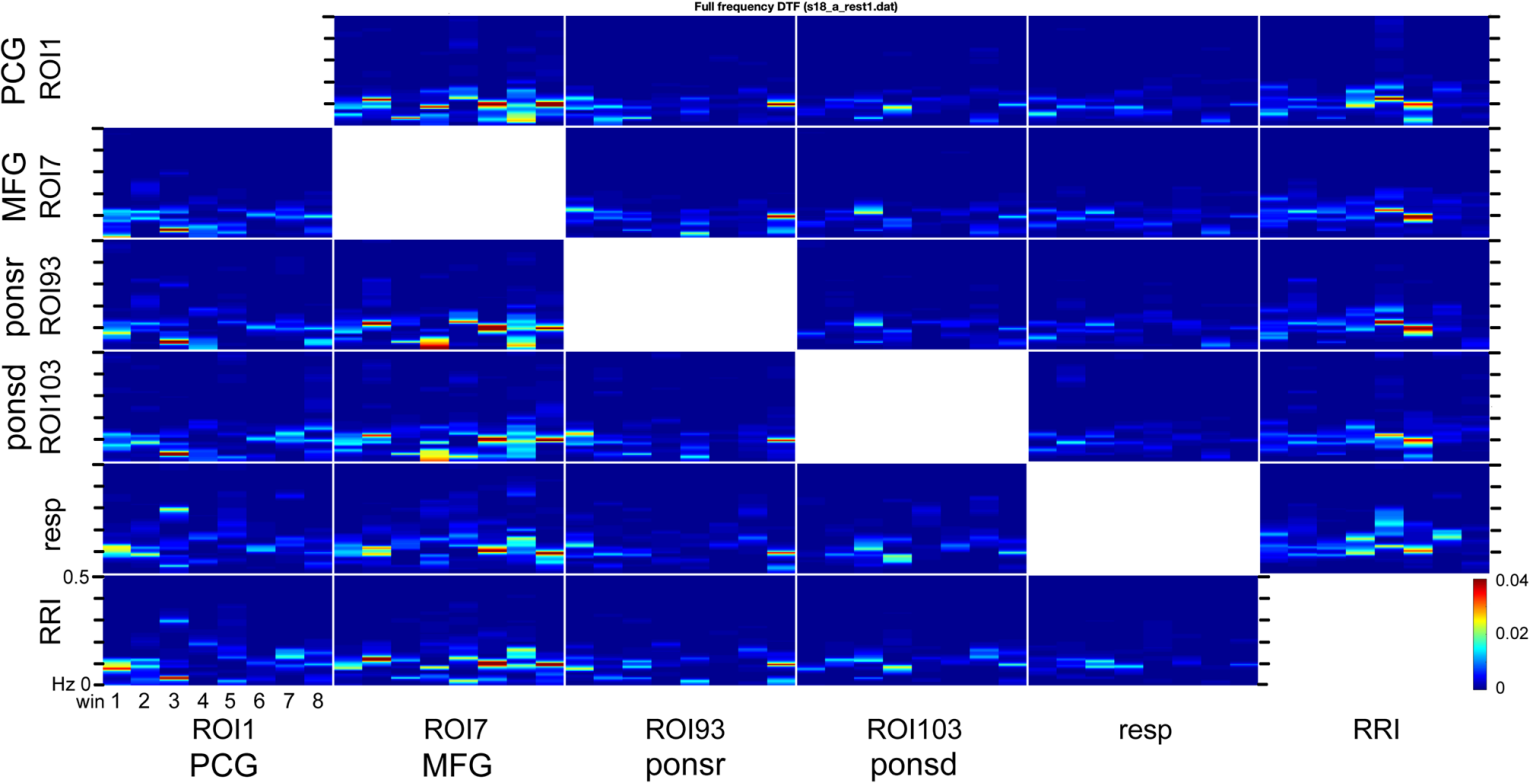
Estimated by means of ffDTF(*f*) information transmissions depending on time and frequency between the 6 channels for an exemplary participant. The transmission of information is from the signal marked below the column to the signal marked at the left. In each box, the ffDTF(*f*) function for eight 40-s long epochs (windows) is shown; *x*-axis: time in seconds, *y*-axis: frequency from 0 to 0.5 Hz. Colors reflect the amplitude size of ffDTF (red indicates the strongest transmission).

The causal coupling between six investigated signals as a function of frequency and time as estimated by means of ffDTF is illustrated in Fig. 2 for an exemplary individual. In each box, the discrete-time axis (horizontal) is divided into 8 non-overlapping epochs. The vertical axis corresponds to frequencies in the 0–0.5 Hz range. Coupling strength is color coded with stronger coupling indicated by hot colors.

**Figure 3:**
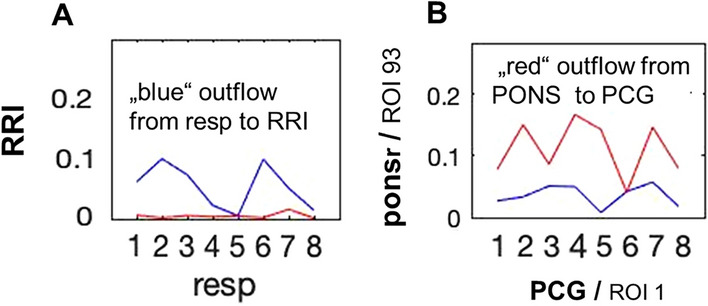
Coupling strength (vertical axis) across 8 time windows (horizontal axis) between RRI and respiratory signal (**A**) for one representative participant in the frequency band of 0.05 to 0.15 Hz; dominant flow from respiration to RRI, characteristic for positive RSA, is documented. Coupling strength between BOLD signals from brainstem rostral pons structure (ponsr, ROI 93) and PCG from another participant (**B**).

For the calculation of ffDTF we used only one filter: 0–0.5 Hz. This was performed for each subject and each resting state. One may observe big variability in time of the obtained ffDTF spectral components (Fig. 2). The strongest transmissions are visible mainly around ~ 0.1 Hz band which corresponds to Mayer waves. We concentrated our attention on this frequency band. The strength of couplings between channels *i* and *j* − *C*_*ij*_ in 0.05–0.15 Hz band was found by integrating ffDTF spectra in this band.

In Fig. 3 the plot at the left (A) shows directed coupling between respiration and RRI. Here, the direction of flow is from the respiratory to the cardiac signal, which is characteristic for the well know respiratory sinus arrhythmia (RSA). The plot at the right (B) shows coupling between two BOLD signals, recorded from the rostral pons (ROI 93) and the PCG. In this example the flow is bi-directional, with a dominant outflow from the brainstem to the PCG in the prefrontal cortex.

### Causal coupling for LA group

**Figure 4:**
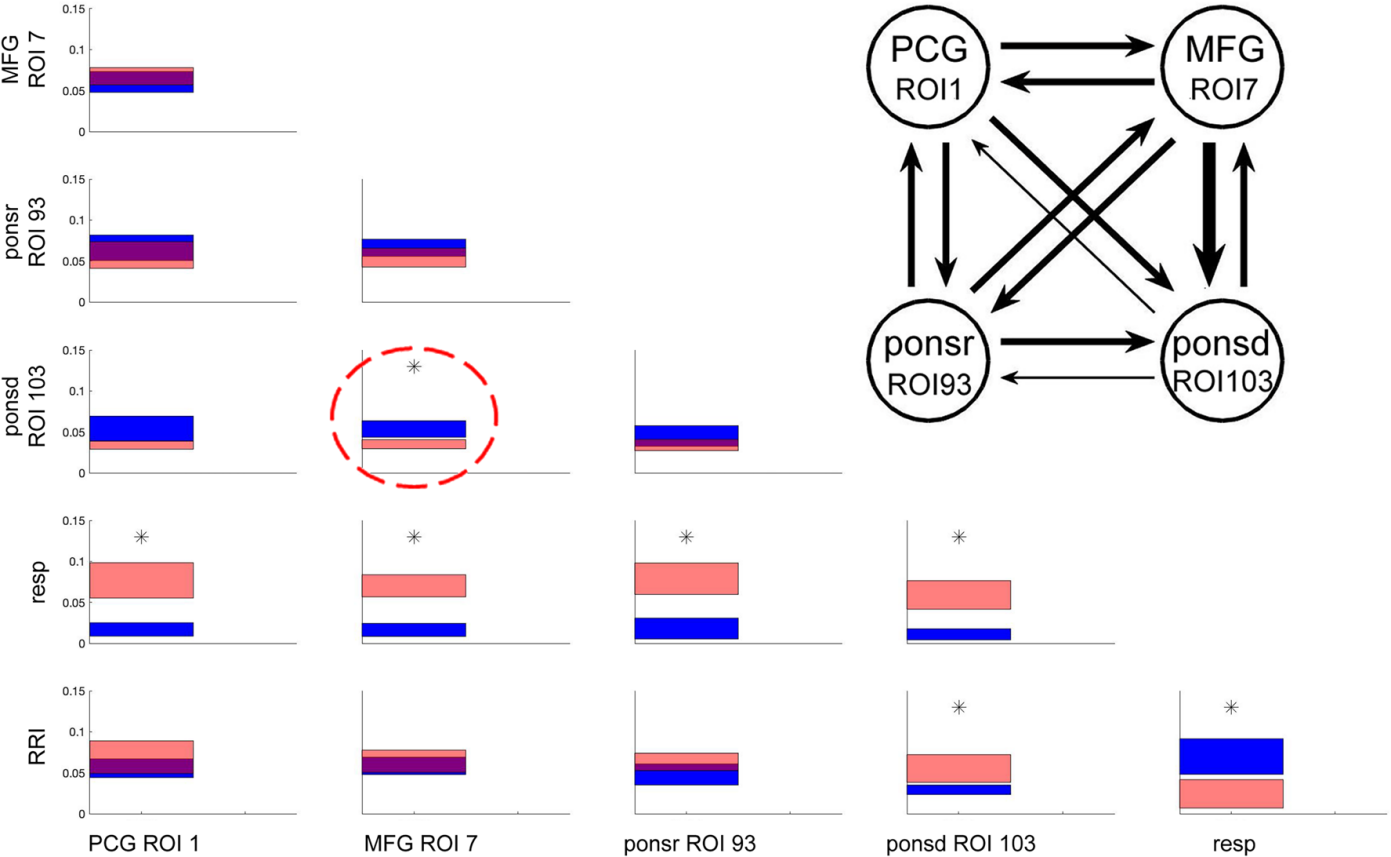
Directed coupling strengths for LA group in 0.05–0.15 Hz frequency band. Each box shows the strength of coupling as a position on the vertical axis. The width of bars is proportional to the mean error. The blue color shows the flow from the signal marked below the given column to the signal marked at the left, and the red color the flow from the signal marked at left to the signal marked below. Significant differences between couplings of inflow and outflow are marked by stars (*p* < 0.05). Note, significant flow from MFG (ROI 7) to caudal pons structure (ponsd, ROI 103) (marked by a stippled circle). The insert shows the coupling strength for BOLD signals only. Each arrow indicates the direction and the strength of information flow.

In Fig. 4, the outflows and inflows between all 6 signals are illustrated. The coupling strengths averaged in time and over subjects are visualized by the position on the vertical axis, and the mean error by the thickness of the bars.

As evidenced by Fig. 4, there was a comparably strong outflow of information from the respiratory system to brain structures and to RRI reflecting the complex cardiocirculatory interaction. The dominant outflow from respiration to the BOLD signal is due to the different signal modalities. The former represents changes of intrathoracic pressure and volume as well as respiratory rate, and the latter subsequent hemodynamic fluctuations delayed from neural signals. The width of bars of the respiratory signal-directed coupling reflects the large variability of breathing patterns across individuals and a massive information outflow from this structure. In contrast, the strength of the BOLD outflow is small, indicating low variability (small standard error) and a minor flow. Note that bi-directional coupling between all BOLD signals is significant only for the flow from MFG (ROI 7) to the brainstem (ROI 103). See insert in Fig. 4 and Table 1.

**Table 1:** Significance of differences between inflows and outflows in terms of percentiles of the distribution obtained by the bootstrap approach (see Methods section) for LA group in the frequency band 0.05–0.15 Hz. Significant differences (marked by bold numbers) are those which exceed the region of 95% of the random distribution of the differences obtained by the bootstrap method. Depending on the sign of the differences between inflows and outflows, they are located below 2.5 and above 97.5 percentile. Flow from the structure marked below the columns to the structures marked beside the rows of the Table.

	PCG ROI1	MFG ROI7	Ponsr ROI93	ponsdROI103	Resp
MFG/ROI7	78.58				
ponsr/ROI93	23.11	6.37			
ponsd/ROI103	3.11	**0.30**	7.32		
resp	**99.97**	**100.00**	**99.97**	**99.98**	
RRI	84.85	73.42	96.42	**99.24**	**0.36**

In Table 1, the significances of differences between outflows and inflows to the given structures are shown for LA in the 0.05–0.15 Hz frequency band.

### Causal coupling for HA group

**Figure 5:**
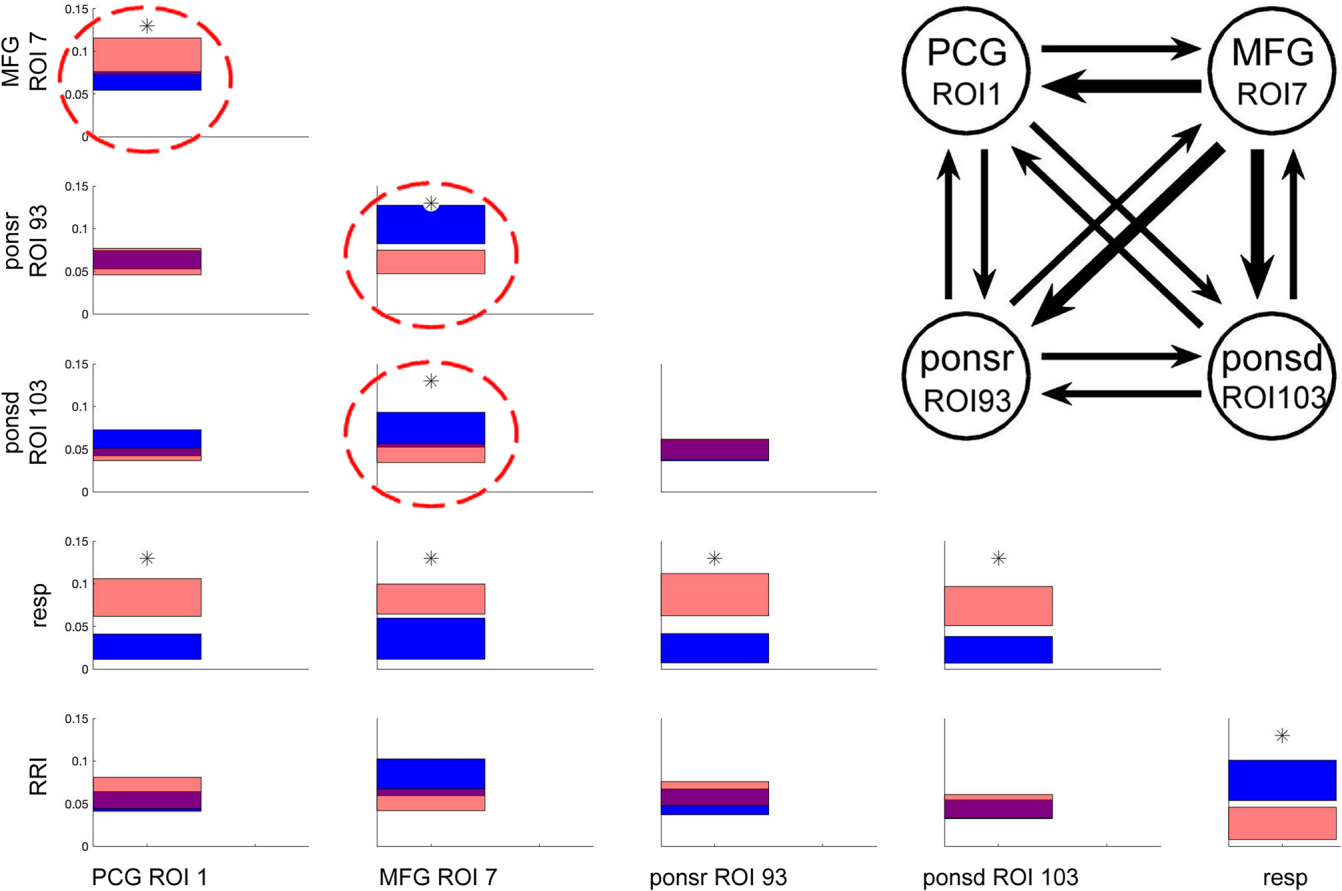
Significant differences between inflows/outflows for HA in 0.05–0.15 Hz band. and insert with coupling strength for BOLD signals only. Note, the strong flow from MFG (ROI 7) to rostral pons (ROI 93) and caudal pons (ROI 103; blue color) and from MFG to the PCG (red color) with significant differences, namely a predominant flow from MFG to PCG, ponsr and ponsd (marked by stippled circles). For further explanations, see Fig. 4.

Directed couplings for high anxiety are shown in Fig. 5. The pattern of findings appears similar to Fig. 4 at the first glance, but there are also important differences. The significance of differences between the in- and outflow to the given structure are shown in Table 2 and also in the insert of Fig. 5. Elevated anxiety showed similar significant changes with respiration and RRI signals (similarly to LA), which could be due to the comparably slight difference in anxiety levels. However, there were significant changes in BOLD signal couplings. First, there was an increased flow from MFG (ROI 7) to the brainstem (caudal pons and rostral pons). Second, a significant flow from MFG (ROI 7) to the PCG (ROI1) can be observed. Remarkably, in the case of coupling between PCG and brain stem (rostral pons) both bi-directional flows are of almost the same strength. This result may provide evidence for the existence of an ascending activation with an origin in the brainstem.

**Table 2:** Differences between in- and outflows in terms of percentiles of the distribution obtained by the bootstrap approach for the HA group. For further explanation, see Table 1.

	PCG ROI1	MFG ROI7	Ponsr ROI93	Ponsd ROI103	Resp
MFG/ROI7	**98.20**				
ponsr/ROI93	39.63	**0.21**			
ponsd/ROI103	7.67	**1.92**	52.61		
resp	**99.93**	**99.71**	**99.92**	**99.80**	
RRI	78.93	3.44	79.59	63.13	**0.37**

Significant differences (marked by bold numbers) are those which exceed the region of 95% of the random distribution of the differences obtained by the bootstrap method. Depending on the sign of the differences between inflows and outflows, they are located below 2.5 and above 97.5 percentile.

### Difference between elevated anxiety (HA) and low/no anxiety (LA)

**Figure 6:**
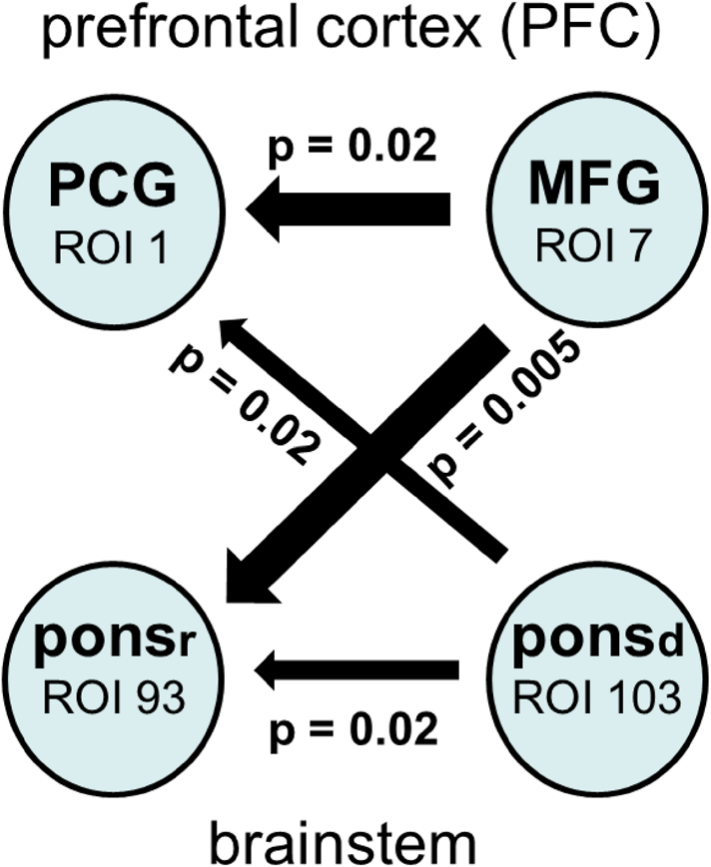
Significant increases in information flow in HA relative to LA. Differences in coupling strength are indicated by the widths of the arrows. Significant changes of the flows are indicated by *p*-values.

In order to distinguish between BOLD signals in both groups, the differences in coupling strengths were analyzed (Fig. 6). Significant changes between BOLD signals are indicated by *p*-values. Remarkable are two findings: the strong outflow from the left MFG (ROI 7) to the left PCG (ROI1) and to the brainstem (rostral pons) and the weak outflow from the brainstem (distal pons, ROI 103) to the PCG and rostral pons (ROI 93). The most significant flow is from MFG to the brainstem during HA.

## Discussion

An analysis of causal coupling during fMRI-induced anxiety in healthy individuals revealed some novel results. In the case of low or no anxiety (LA), the information flow from the MFG to the brainstem was increased. During elevated anxiety (HA), an increased flow from the brainstem to PCG was observed in addition to the increased top-down flow.

### Anxiety, RSA and BOLD signals in brain stem

The brain stem is a highly complex, densely packed structure of small sized regions and the location of one major cerebral artery (basilar artery). Furthermore, it is in proximity to tissues leading to image distortion. Therefore, the disentangling of signals from physiological noise using techniques such as RETROICOR^28^ and independent component analyses are highly recommended^29^. We recorded BOLD signals from the brainstem (without applying any of the correction methods), simultaneously with the respiratory signal by using a chest belt, and the cardiac beat-to-beat intervals recorded from precordial electrocardiographic leads. From the ROIs only such with a number of voxels > 1000 were used. Importantly, the respiratory BOLD artefact is present and constitutes an important marker for neuro-BOLD coupling in the brainstem. Interestingly, without any preprocessing of BOLD signals side-differences were found in the brainstem, with a dominance of respiratory BOLD artefacts in the right side and BOLD signals in the left side^9^. This was the reason that for our study only BOLD signals from the left hemisphere and left brainstem, respectively, were used. Remarkably, Lambert et al.^30^ used a specific segmentation algorithm, high-resolution data and multivariate modelling and found significant brainstem asymmetries. Whether a link exists between this asymmetry and the asymmetry in slow BOLD signals requires further research.

In a quiet and anxiety-free state situation, particularly accompanied by emotional well-being^31^, respiratory sinus arrhythmia (RSA) is a common phenomenon. Typically, inspiration is accompanied by acceleration of the cardiac cycle and expiration is associated with deceleration (also called “positive RSA”). The brainstem is also important for anxiety processing. Notably, in situations of elevated anxiety, negative RSA (i.e., cardiac deceleration during inspiration, acceleration during expiration^32^) is prevailing^33^. This negative RSA, which was primarily observed in frequency bands centered at 0.1 and 0.15 Hz^33^ seems to be accompanied by two BOLD signals in brain stem. One is a respiratory BOLD artefact induced by vessel motion of the basilar artery (located directly in the brainstem) leading the respiratory signal by ~ 0.3 s. The other BOLD signal is lagging the respiratory BOLD artefact by ~ 2.5 s ^34^ and can be interpreted as a hemodynamic equivalent (response) of the rhythmic neural signal which is responsible for the generation of the respiratory signal that is time-locked to (coherent with) the cardiac signal (reflecting negative RSA). Remarkably, the delay of 2.5 s between these two BOLD signals in the human brainstem corresponds nearly perfectly with the time shift between slow fluctuation (~ 0.1 Hz) of gamma power and BOLD oscillations in mice of 2.6 s^35^. This provides strong evidence that this second BOLD is of neural origin (neural BOLD) with a source in the brainstem. Support for such a time span of 2.5 s also comes from the coherent timing between vascular and neural BOLD components, with the former preceding the later by ~ 2.5 s^5,36^.

### Information flow from middle frontal gyrus to brainstem (top-down activation)

Breathing is not only controlled by metabolic demands but also is affected by anxiety and other emotional changes^37^. In a study on healthy young men sitting in a chair in a quiet room, HR, state anxiety, and respiratory data were measured^38^. Even in this situation with quiet breathing, elevated anxiety led to an increased respiratory rate with a presumed central control. However, during HA and LA, while being exposed to noisy MRI scanner in a small ‘tunnel’, a strong information flow from the MFG to the brainstem could be observed. It should be noted that noradrenalin and serotonin are both important for anxiety processing^39^. The former is controlled by the locus coeruleus (LC) in the tegmentum of the pons^39^ and the latter by the raphe nuclei distributed across the brainstem with close neuronal‐vascular relationships of both neuron groups^40^.

Anxiety is associated with generally increased LC activity that is not oscillatory, but sustained^41^. Totah et al.^42^ reported on transient increases in PFC gamma power that preceded peaks in the 5-Hz LC multi-unit activity. Note, that the differences between animal experiments and findings with humans may limit the comparability of the results^43^. During LA, the flow from the MFG to the caudal pons structure (ROI 103) was dominant. In contrast, the flow from the MFG to the brain stem was not only increased in HA but also spread to rostral pons structures (ROI 93) and in addition also to the PCG. This anxiety decrease between HA and LA is accompanied by a decrease of the flow from MFG to brainstem and should be also associated with a lower LC activation.

### Information flow from respiration to BOLD signals

The information flow from the respiratory to all BOLD signals is similar for LA (Fig. 4) and HA (Fig. 5). It should be noted that BOLD signals have a hemodynamic origin and are always delayed relative to the neural signals (exhibiting neurovascular coupling, e.g.: Arthus & Bonface^44^), which might explain the similarity of these findings The location of respiratory centres (central pattern generator) are integrated into a spatially distributed pontine–medullary respiratory network and includes also the preBötzinger complex^45^. Although the BOLD signal recorded at ROI 103 in the caudal pons structure is in close vicinity to the respiratory centres, this signal must not be considered representative for the neural drive of respiratory activity. Hence, a time delay of a few seconds is not only observed between gamma power and BOLD oscillation of ~ 0.1 Hz frequency^35^, slow alpha/beta EEG and deoxyhemoglobin concentration oscillations^27^, or slow RRI and BOLD oscillations, but also between respiration and BOLD signals. Remarkably, the flow from respiratory to BOLD signals and from respiratory to RRI signals was about the same for LA and HA, thus suggesting that both flows represent fundamental physiological phenomena, namely neurovascular coupling and RSA^33^.

Concerning the respiration rate, the work of Lambertz & Langhorst^46^ and Perlitz et al.^47^ concerning the “0.15-Hz rhythm” in the brainstem is especially important. These authors reported rhythmic periods of spindle waves in reticular neurons, which are phase synchronized relative to respiration with a frequency ratio of 1:1, 1:2, and 1:3. The relationship between the 0.15-Hz rhythm reflecting a pacemaker-like oscillatory activity with an origin in the brainstem and the above-mentioned breathing pattern is a common observation. Whether the BOLD oscillations in the brainstem close to the respiratory centres (e.g., ROI 103) are associated with the neural activity from respiratory neurons or originated from a slow pacemaker-like oscillatory activity remains an open question.

### Information flow from the brainstem to the pre-central gyrus (ascending activation)

Figure 6 shows the most dominant differences in coupling between HA and LA with the corresponding *p*-values. In addition to the increased information flow from the MFG to the brainstem, the flow from the brainstem to the PCG is particularly noteworthy. This flow from the brainstem to the left PCG activates an important part of the somatomotor network (SMN)^48^. Further activation of this SMN results from the increased flow from the MFG. The SMN and the salience network (SN) display increased functional network connectivity in AD together with a decrease in serotonergic and noradrenergic activity. Whether the neurotransmitter are associated with increased or decreased network connectivity is also evident during transient elevated anxiety in healthy individuals is not known. It should be noted, however, that there seems to be an increased flow from the MFG to pontine structures with the locus coeruleus (LC) as an important hub for the control of the noradrenergic system^39^.

The increased flow into the PFG as part of the somatomotor network from both the MFG and the brainstem underlines the importance of these neural structures for anxiety control. While the descending flow into the brainstem was predominant during HA, the ascending flow was small and restricted to the caudal pons (ROI 103), as revealed by the calculation of flow differences between HA and LA (Fig. 6). Inspection of the coupling strengths for LA (Fig. 4) and HA (Fig. 5) showed not only a significant inflow into PFC but also a dominant outflow from the brainstem (ROI 93) to PFC for HA, which, however, was not significant, This observation can be seen as confirmation of an outflow of information not only from ROI 103 but also from ROI 93, both located in close vicinity to the pons.

The significantly larger descending than ascending flow with an origin in the brainstem is an interesting result, which indicates, besides the hypothesized pacemaker-like neural activity in the brainstem, presumably a further mechanism for successful anxiety processing. Such a mechanism may be due to an enhanced cerebral blood flow in the baroreflex loop in connection with an increase of vascular BOLD oscillations.

While the examined structures in the PFC are clearly defined, the brainstem structures are more complex. One reason is the location of different systems and/or the multiple integrative functions of the brainstem. Other reasons concern problems with recordings of BOLD signals of sufficient quality due to their small size and possible image distortion^49^. These findings indicate, that the neural source of slow BOLD oscillations is not a specific locus in the sense of cell groups but may be a distributed network similar to the reticular neurons responsible for the “0.15-Hz rhythm”^47^. Whereas resting-state activity with dominant slow vascular BOLD components (*f* < 0.1 Hz) characterizes relatively anxiety-free situations^50^ the existence of slow neural and vascular BOLD components, the latter leading the former, was discussed by Pfurtscheller et al.^7,36^ in healthy subjects during fMRI-related anxiety and by Tumati et al. in connection with AD.

Summarizing, HA and LA can be distinguished by the strength of information flow from the MFG to the brainstem during HA and also by the outflow from the brainstem to the PCG (representing neural BOLD components). The latter is most likely associated with a pacemaker-like activity source in reticular brainstem neurons. Further research is necessary, especially to investigate the ascending information flow in subjects with ”non-successful” anxiety processing and elevated resting-state anxiety.
